# Supplementary material for: Variation of Deoxynivalenol Levels in Corn and Its Products Available in Retail Markets of Punjab, Pakistan, and Estimation of Risk Assessment
Source: Toxins (Basel). 2021 Apr 22;13(5):296. doi: 10.3390/toxins13050296 (PMC8143574; doi:10.3390/toxins13050296)
Supplement: Supplementary file 1 [file toxins-13-00296-s001.zip › toxins-1111627-supplementary.pdf]

Supplementary Materials: Variation of Deoxynivalenol Levels in Corn and Its Products Available in Retail Markets of Punjab, Pakistan, and Estimation of Risk Assessment

Shahzad Zafar Iqbal, Ahmad Faizal Abdull Razis, Sunusi Usman, Nada Basheir Ali and Muhammad Rafique Asi

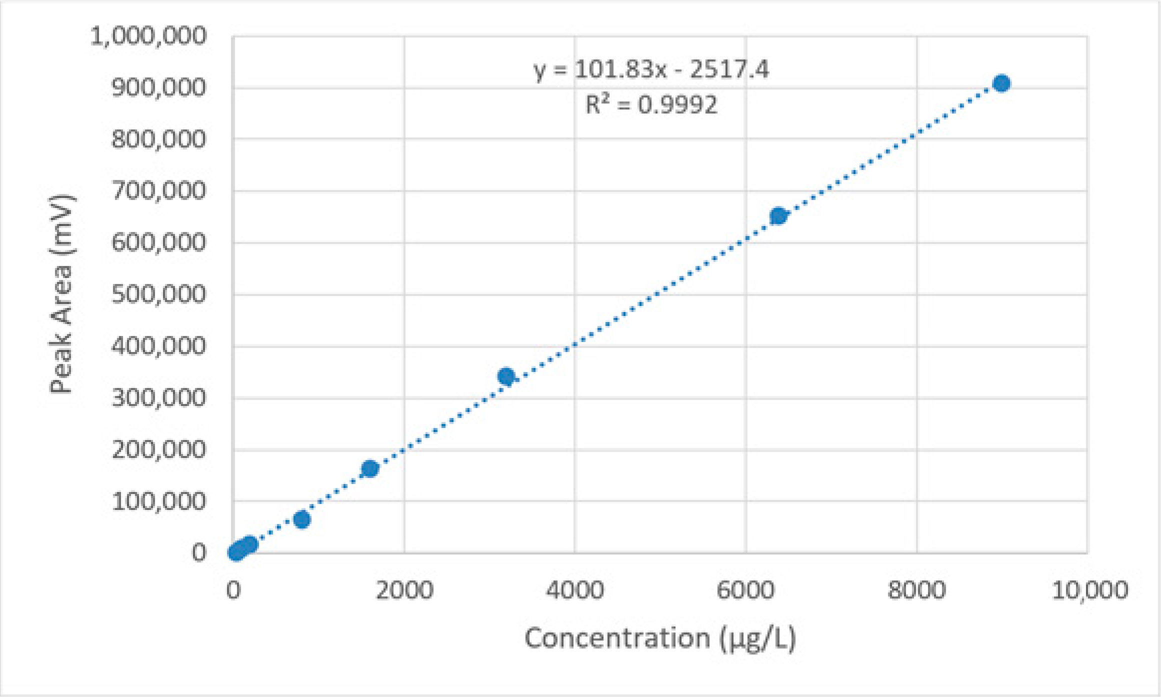

Figure S1. The standard curve of DON.
